# Management of infection by the Zika virus

**DOI:** 10.1186/s12941-016-0172-y

**Published:** 2016-09-29

**Authors:** Melissa Barreto Falcao, Sergio Cimerman, Kleber Giovanni Luz, Alberto Chebabo, Helena Andrade Brigido, Iza Maria Lobo, Artur Timerman, Rodrigo Nogueira Angerami, Clovis Arns da Cunha, Helio Arthur Bacha, Jesse Reis Alves, Alexandre Naime Barbosa, Ralcyon Francis Teixeira, Leonardo Weissmann, Priscila Rosalba Oliveira, Marco Antonio Cyrillo, Antonio Carlos Bandeira

**Affiliations:** 1 Universidade Estadual de Feira de Santana, Avenida Transnordestina, s/n, Feira de Santana, BA CEP 44036-900 Brazil; 2 Instituto de Infectologia Emilio Ribas, Avenida Doutor Arnaldo, 165, São Paulo, SP CEP 01246-000 Brazil; 3 Universidade Federal do Rio Grande do Norte, Rua Conego Monte, s/n, Natal, RN CEP 59037-170 Brazil; 4 Universidade Federal do Rio de Janeiro, Avenida Professor Rodolpho Paulo Rocco, 255, 50. andar, Rio de Janeiro, RJ CEP 21941-913 Brazil; 5 Universidade Federal do Para, Rua dos Mundurucus, s/n, Belem, PA CEP 66060-060 Brazil; 6 Universidade Federal de Sergipe, Avenida Claudio Batista, s/n, 3o. andar, Aracaju, SE CEP 49060-100 Brazil; 7 Hospital Professor Edmundo Vasconcelos, Rua Borges Lagoa, 1450, Sao Paulo, SP CEP 04038-905 Brazil; 8 Universidade Estadual de Campinas, Rua Alexander Fleming, 181, Campinas, SP CEP 13083-970 Brazil; 9 Universidade Federal do Parana, Rua General Carneiro, 181, Curitiba, PR CEP 80060-900 Brazil; 10 Hospital Israelita Albert Einstein, Avenida Albert Einstein, 627, Bloco A1, Sala 220, Sao Paulo, SP CEP 05651-901 Brazil; 11 Universidade Estadual Paulista Julio de Mesquita Filho, Distrito de Rubiao Jr, s/n, Botucatu, SP CEP 18618-970 Brazil; 12 Universidade de São Paulo, Rua Doutor Ovidio Pires de Campos, 333, Sao Paulo, SP CEP 05403-010 Brazil; 13 Hospital do Servidor Publico Municipal, Rua Castro Alves, 60, São Paulo, SP CEP 01532-000 Brazil; 14 Hospital Aliança, R. Juracy Magalhães Júnior, 2096, Salvador, BA CEP 41920-000 Brazil

**Keywords:** Zika virus infection, Guideline, Diagnosis, Therapeutics

## Abstract

A panel of national experts was convened by the Brazilian Infectious Diseases Society in order to organize the national recommendations for the management of zika virus infection. The focus of this document is the diagnosis, both clinical and laboratorial, and appropriate treatment of the diverse manifestations of this infection, ranging from acute mild disease to Guillain-Barré syndrome and also microcephaly and congenital malformations.

## Background

The Zika virus is an arbovirus of the genus *Flavivirus*, in the family Flaviviridae, which was first identified in 1947, in the Zika Forest in Uganda during a monitoring program on wild yellow fever [1–4]. It is related to other flaviviruses, including the viruses that cause dengue, yellow fever and West Nile fever.

Outbreaks of the disease were first notified in the Pacific region in 2007 and 2013, respectively in the Yap islands and in French Polynesia, and then in the Americas (Brazil and Colombia) and in Africa (Cape Verde) in 2015 [2, 3, 5, 6]. Rapid geographical expansion has been observed since then, with 40 countries in the Americas reporting autochthonous transmission as sporadic cases or outbreaks. It is also important to mention the growing number of countries on other continents that have been notifying occurrences of imported cases of Zika virus infection, thus demonstrating its great potential for dissemination on a worldwide scale [7].

Through occurrences of Zika outbreaks, the central nervous system and autoimmune complications that were previously reported in French Polynesia have also come to be observed in the Americas.

Zika infection during pregnancy has been correlated with congenital microcephaly, fetal malformations and fetal losses. This led the Brazilian Ministry of Health to declare a state of public health emergency of national importance in November 2015, after observation of changes to the epidemiological pattern of occurrences of microcephaly in Pernambuco and other states in northeastern Brazil [8, 9].

In the light of the significant increase in the incidence of neurological syndromes and cases of microcephaly that were potentially related to the Zika virus, the World Health Organization (WHO) declared an international public health state of emergency in February 2016. To put the importance of this event into context, this was the fourth time that WHO has ever declared a worldwide state of emergency in relation to a viral epidemic. The previous decisions were made in relation to H1N1 (2009), poliomyelitis (2014) and Ebola (2014).

On February 18, 2016, the Brazilian Ministry of Health issued an ordinance that made it compulsory to notify suspected cases of Zika throughout the country. This disease has thus been added to other arboviruses, such as dengue, yellow fever, West Nile fever and chikungunya, which were already on the national list of diseases with compulsory notification [10].

## Epidemiology

### Geographical distribution

In humans, the Zika virus was first identified in Uganda and Tanzania, in 1952 [11]. Between 1952 and 1981, a variety of serological evidence regarding infection by this virus was reported from countries in Africa and some parts of Asia [4].

The first epidemic outside of Africa and Asia occurred in 2007, in the Yap islands of Micronesia. It was estimated that more than 70 % of the population over the age of 3 years became infected [2]. Another large outbreak of Zika fever occurred concomitantly with a dengue epidemic (serotypes 1 and 3) in French Polynesia in 2013–2014, affecting around 32,000 people [3].

In 2014, cases of Zika virus infection were reported on Easter Island, which is Chilean territory [12]. In May 2015, some months after reports of increased incidence of exanthematous febrile disease in states of northeastern Brazil, which until then were of unidentified cause, presence of Zika virus circulation was confirmed in this country. This was initially confirmed in Bahia on April 29, 2015, from analysis on samples from patients with an exanthematous condition in Camaçari, Bahia, and subsequently in Rio Grande do Norte on May 9, 2015, with identification of the Asian genotype [13, 14]. Also in May, cases in Sumaré and Campinas (São Paulo), Maceió (Alagoas) and Belém (Pará) were confirmed through laboratory tests. Since then, rapid expansion of the areas of circulation and autochthonous transmission of the virus has been observed, notably in states of the northeastern region of Brazil. It is estimated that more than one million Brazilians became infected with the Zika virus in 2015, thus reflecting the capacity of the virus to cause large-scale outbreaks in places where the biological vector is present.

Worldwide, the virus is now circulating in 65 countries and territories, mostly in the Americas [15].

### Transmission methods

Zika is transmitted primarily through the bites of infected mosquitos of the genus *Aedes*, especially *Aedes aegypti* and *Aedes albopictus* [16].

In humans, except for pregnant women, the period of viremia is short and it is most frequently identified by the 5th day after the symptoms start. The RNA of the Zika virus has been identified in blood as early as on the 1st day, and also only up to 11 days after the disease begins. Prolonged Zika virus RNA was detected in serum of four symptomatic pregnant women in up to 46 days after symptoms onset and in one asymptomatic pregnant woman 53 days after infection [17]. In pregnant women Zika virus RNA has been detected for up to 10 weeks after infection [18].

In addition to vector transmission, other forms of transmission that so far are just theoretical or anecdotal have started to receive greater attention. The RNA of the Zika virus has now been detected in blood, urine, semen, saliva, female genital tract secretions, cerebrospinal fluid, amniotic fluid and breastmilk [16, 19– 25].

After two likely cases of transmission through blood transfusion in Campinas, São Paulo, were identified, there has been much discussion about the importance of this transmission route [26].

Reports of detection of Zika in urine through PCR up to 20 days after the start of symptoms, in a study conducted in French Polynesia, even without any confirmation of infectiveness, have given rise to discussion on the need for better comprehension of the importance of this biological material as an infecting agent [21].

Zika has been detected in semen for periods of up to 10 weeks after recovery from the symptoms of the infection, and likely cases of sexual transmission from men to women have been described. Eleven countries have reported evidence of person-to-person transmission of Zika virus, probably via a sexual route [15].

Tests conducted on the amniotic fluid of pregnant women with possible Zika virus infection whose fetuses had been diagnosed as presenting microcephaly have been found to be positive for the Zika virus [27]. This shows that the virus has the capacity to cross the placental barrier and, increasingly evidently, that it causes fetal malformations.

Presence of the virus has already been shown through detection of viral RNA in the breastmilk of mothers with conditions of acute infection. It is expected a higher viral load of Zika virus in maternal milk for women infected near delivery, and not expected to occur with first trimester infections. In New Caledonia it has been reported the presence of infectious Zika virus particles in breast milk with substantial viral loads [28]. However, since there have not been any confirmed cases of transmission through breastfeeding, the guidance continues to be that breastfeeding should be maintained, given that the benefits of breastfeeding override the risks of virus transmission through breastmilk, which remains unproven [22]. In the light of present knowledge, identification of the virus in urine, breastmilk, saliva and semen is potentially useful in diagnosing the disease, but the possibility of importance of such findings for virus transmission to other people cannot be confirmed.

## Clinical manifestations

### Signs and symptoms

It has been estimated that clinical manifestations occur in around 20 % of infected individuals. Hence, asymptomatic infection occurs more frequently. These estimates have been based on a single study that was conducted through a household serological survey involving serological tests for Zika (IgM) [2].

The duration of incubation in humans is unknown, but it has been estimated to be 2–14 days after the bite of the vector mosquito [29].

The clinical condition typically includes a maculopapular rash, frequently accompanied by pruritus, low fever (37.8–38.5 °C), arthralgia (especially in the joints of the hands and feet) and non-purulent conjunctivitis. Other manifestations that have commonly been reported include myalgia, headache, retro-orbital pain and asthenia. Periarticular edema, oral lymphadenopathy, oral ulcers, abdominal pain, nausea and diarrhea may also occur [2, 13, 14].

In most patients, the symptoms are usually mild and present spontaneous resolution after 2–7 days. However, in some patients, arthralgia may persist for around 1 month.

- So far, the length of the immunity conferred through natural infection with the Zika virus remains unknown.

*Brazilian ministry of health’s definition of suspected zika cases*

- Suspected case: “Patients who present pruriginous maculopapular exanthema accompanied by two or more of the following signs and symptoms:
  - Fever
  - Conjunctival hyperemia without secretion and pruritus
  - Polyarthralgia
  - Periarticular edema”

*Brazilian ministry of health’s definition of confirmed zika cases*

- Confirmed case: “Suspected case in which one of the following tests is positive or shows a specific reaction for a diagnosis of Zika:
  - Isolation of the virus
  - Detection of viral RNA through the reverse transcriptase reaction (RT-PCR)
  - IgM serological test (in populations presenting co-circulation of the dengue virus, there is a high chance that false positive reactions may occur)”
  - For epidemiological control after confirmation of autochthonous circulation, other acute cases of Zika should be confirmed through clinical-epidemiological criteria, except when in pregnant women or in situations of neurological manifestations and death.

### Congenital complications

Congenital malformations, including microcephaly, generally have complex multifactorial etiology and may have been caused through infection during pregnancy or through chromosomal disorders, exposure to environmental toxins or metabolic diseases, as shown on Table 1. The temporal and spatial relationship between Zika outbreaks and higher incidence of microcephaly in states with documented autochthonous transmission has started to indicate that the existence of a causal relationship between these two epidemiological events is increasingly likely [30].

**Table 1:** Etiological agents and risk factors for microcephaly

Period and types	Etiology
Congenital	
Genetic	Inherited genetic disorders, syndromes and mutations
External, chemical agents	Brain injury due to teratogenic drugs, toxins and chemical products, including foetal alcohol syndrome, radiation
Metabolic diseases	Diabetes
Nutritional	Malnutrition (maternal malnutrition, maternal folate deficiency, placental insufficiency)
Vascular	Hypoxic-ischemic lesions
Infectious	Transplacental infections of the central nervous system STORCH infections; syphilis, toxoplasmosis, rubella, cytomegalovirus, herpes simplex virus, HIV other viruses
*Post-partum*	
Brain vascular and non-vascular injuries Meningitis Encephalitis Congenital encephalitis due to HIV Cupper intoxication Chronic renal failure	

*Source* [31]

This relationship has become increasingly consistent, consequent to detection of viral RNA using the PCR technique for Zika in amniotic fluid, placenta, umbilical cord blood and cerebral tissue. The capacity of the virus to infect and cross the placental barrier such that it might then affect the nerve tissue during its formation has also been demonstrated [32, 33].

In Brazil, since the confirmation of the Zika outbreak, the incidence of microcephaly has become more than 20 times higher than what would otherwise have been expected [34].

During an investigation conducted in relation to 35 children with microcephaly, 74 % of the mothers in northeastern Brazil who were suspected of having had Zika during their pregnancies reported that they had had a skin rash during the first and second trimesters [35].

Retrospectively, after Brazil’s notification of cases to WHO, cases of microcephaly were also identified in French Polynesia. These cases have recently been reported in the literature [36].

According to guidance from the Ministry of Health, it is compulsory to notify cases of fetuses that are identified as presenting central nervous system abnormalities during pregnancy.

In April 2016, the Centers for Diseases Control and Prevention (a public body in the United States for research, statistics, control and prevention of diseases) concluded that there was a causal relationship between prenatal infection by the Zika virus and microcephaly other cerebral abnormalities. This conclusion was based on evidence regarding Zika virus infection during prenatal development that was consistent with the defects observed, with occurrence of a rare and specific phenotype involving microcephaly and cerebral abnormalities in fetuses or newborns with confirmed or presumed congenital infection due to the Zika virus, and on data that strongly supported biological plausibility, including identification of the Zika virus in the cerebral tissue of the fetuses and babies affected [37].

## Case definition

### Notified case

“A fetus that presents at least one of the following criteria relating to abnormalities of the central nervous system, as identified through ultrasound examination [9]:

- Presence of cerebral calcifications AND/OR
- Presence of ventricular abnormalities AND/OR
- At least two of the following signs of abnormalities of the posterior fossa: hypoplasia of the cerebellum, hypoplasia of the cerebellar vermis, widening of the posterior fossa greater than 10 mm and agenesis/hypoplasia of the corpus callosum.”

*The findings from transfontanelle ultrasound and/or cranial tomography include* [27, 35, 38, 39]:

- Cerebral calcifications, especially periventricular, in the parenchyma, thalamic areas and basal ganglion.
- Ventriculomegaly.
- Lissencephaly.
- Hypoplasia of the brain stem and cerebellum
- Abnormality of white matter attenuation.

*Other ultrasound findings*

- Arthrogryposis.
- Intrauterine growth retardation.
- Arterial flow abnormalities in the cerebral or umbilical arteries.
- Oligohydramnios or anhydramnios.

- ❖ Ultrasound findings can be detected from the 18th to 20th week of pregnancy onwards.

There may be ocular involvement, especially pigment abnormality and macular atrophy, and also abnormalities of the optic nerve [40, 41].

### Complications of the central nervous system

Guillain-Barré syndrome (GBS) is an autoimmune disease characterized by acute inflammatory demyelinating polyradiculoneuropathy. Motor function is usually affected, starting distally and progressing proximally over a 4-week period. The patients present generalized weakness, areflexia and varying degrees of sensory disorders and disorders of cranial nerve involvement. Its forms range from those with both motor and sensory impairment to those solely with sensory impairment. The risk increases with age and it occurs more frequently among men than among women. Approximately 25 % of the patients require admission to an intensive care unit and 3–5 % die. The expected annual incidence is one case per 100,000 inhabitants [42].

Higher incidence of GBS was observed concomitantly with the Zika outbreaks in French Polynesia, Brazil, El Salvador, Colombia, Suriname and Venezuela [43].

A retrospective case–control study conducted in French Polynesia and published in February 2016 reported on 42 cases of GBS that occurred during the outbreak of 2013–2014, with detection of IgM or IgG for Zika in 98 % of the patients and neutralizing antibodies for Zika in 100 % of the cases, compared with 56 % of the control group. The most common symptoms were generalized muscle weakness (74 %) and facial paralysis (64 %) [44].

Bilateral facial paralysis was also frequently observed. Most of the patients (88 %) reported that, on average 6 days before the start of neurological symptoms, they had a condition that was compatible with Zika virus [44].

In Venezuela, 252 cases of GBS were notified between January 1 and 31, 2016, with PCR positive for Zika in three cases, including one fatal case [45].

In Brazil between January and November 2015, 1708 cases of GBS were notified. This total reflected very significant increases in the numbers of cases in some states, especially Alagoas (516.7 %), Bahia (196.1 %), Rio Grande do Norte (108.7 %), Piauí (108.3 %), Espirito Santo (78.6 %) and Rio de Janeiro (60.9 %) [46].

So far, the determinants relating to the increased incidence of GBS in Brazil, Colombia, El Salvador and Suriname have not been definitively established, especially with regard to the scenario of simultaneous circulation of dengue, chikungunya and Zika, which are all potentially related to occurrences of neurological syndromes [43].

Other arbovirus diseases such as dengue, chikungunya, Japanese encephalitis and West Nile fever have already been incriminated as agents related to occurrences of GBS.

Just like other flaviviruses, the Zika virus may cause other neurological syndromes such as meningitis, meningoencephalitis and myelitis, as described in the outbreak in French Polynesia [44].

## Laboratory abnormalities

The laboratory abnormalities are nonspecific. There have been reports of mild to moderate leukopenia and thrombocytopenia, and slight elevation of assayed concentrations of serum lactic dehydrogenase, gamma glutamyl transferase and markers of inflammatory activity (proteins, fibrinogen and ferritin) [8].

Because of the scarcity of clinical studies, what has been described are slight abnormalities in hematological parameters and even smaller changes in liver enzymes. In general, there are no significant abnormalities in these parameters.

## Differential diagnosis

- Dengue: higher fever and greater severity of myalgia and asthenia. There may be complications due to hemorrhage and hemodynamic abnormalities, including shock. Does not usually cause conjunctivitis. Significant abnormalities in laboratory tests, with hemoconcentration, low platelet count and liver enzyme abnormalities.
- Chikungunya: high fever as observed in dengue, but with the major differential that the polyarthralgia/polyarthritis starts suddenly in a severe and debilitating manner. This may be associated with articular/periarticular edema from the outset of the condition. Pruriginous skin rash may be present, with a duration that is usually shorter than in Zika. It may follow a course that includes mild conjunctivitis.

The main differential features of dengue, chikungunya and zika virus infection are shown on Table 2.

- Parvovirus: may cause acute symmetrical arthritis or arthralgia, most frequently in the small joints of the hands and feet, and in the wrists and knees. Skin rash is frequently observed.
- Rubella: generally causes low fever and coryza. Presence of skin rash initially of the face and then spreading to the trunk. There may be arthritis and lymphadenopathy.
- Measles: presence of fever, coughing, sore throat, coryza, conjunctivitis and lymphadenitis. Koplik spots may precede the generalized rash.
- Rickettsiosis: characterized by occurrence of fever, headache, myalgia and centripetal non-pruriginous maculopapular exanthema. The complications have included hemorrhagic suffusion, hemorrhagic, respiratory insufficiency, kidney failure, neurological abnormalities and shock.
- Malaria: periodic fever, paroxysm, kidney failure, jaundice, altered levels of consciousness, hepatomegaly or splenomegaly and history of exposure in transmission areas.
- Leptospirosis: severe myalgia, ocular suffusion, rubinic jaundice, oliguria and subconjunctival hemorrhage. History of exposure to contaminated water.

**Table 2:** Differential diagnosis of dengue, zika and chikungunya in symptomatic cases

Signs/symptoms	Dengue	Zika	Chikungunya
Fever (duration)	Higher than 38 °C (4–7 days)	No fever or low fever < 38.5 °C (1–2 days)	High fever > 38 °C (2–3 days)
Skin rash (frequency)	Moderately elevated	Elevated	Moderately elevated
Myalgia (frequency)	+++/+++	++/+++	++/+++
Arthralgia (frequency)	Rare	Variable, in wrists and hands, with complete regression	Frequent and in multiple joints
Severity of arthralgia	Mild	Mild/moderate	Moderate/severe
Articular/periarticular edema (frequency)	Rare	Mild severity	Frequent and moderate to severe
Non-purulent conjunctivitis	Rare	50–90 % of cases	30 %
Headache (frequency and intensity)	Frequent and high intensity	Frequent and moderate intensity	Frequent and moderate intensity
Pruritus	Mild	Moderate to severe	Mild
Lymphadenopathy (frequency)	Rare	Moderate	Moderate
Neurological complications	Encephalitis (rare)	Guillain-Barré syndrome, encephalitis (rare)	Guillain-Barré syndrome, encephalitis (predominantly in neonates)

Adapted from a panel of specialists of the Brazilian Society of Infectology (SBI), as modified from the source: Health Surveillance Secretariat of the Ministry of Health “Surveillance and response protocol for occurences of microcephaly related to zika virus infection” Brasilia, Federal District, December 2015

Many recent publications have emphasized the occurrence of dual infections, including Zika and Dengue, and Zika and Chikungunya co-infections [47–51]. With the ongoing circulation of these viruses and the relatively similar clinical presentations it is urgently needed the availability of molecular platforms for a more precise diagnosis.

## Laboratory diagnosis

### Types of laboratory samples available and samples required

The specific laboratory diagnosis is based mainly on detection of viral RNA from clinical specimens. In blood samples, detection is possible for a period of 1–5 days after the start of symptoms [52, 53]. Negative results do not rule out the diagnosis, because the sensitivity of RT-PCR is estimated to be 40 %.

Because of the greater persistence of the virus in urine, patients seen after the fifth day of the disease should undergo RT-PCR on urine. This analysis is indicated up to the 15th day after the start of symptoms [52, 53].

It is generally considered that serological tests are able to detect IgM from the 4th day and IgG from the 12th day.

Serological tests for Zika in populations with simultaneous or previous circulation of other flaviviruses may be imprecise because of the risk of cross-reactions, thereby leading to false positive results. For this reason, positive results should be analyzed cautiously since they might represent previous exposure to other flaviviruses (such as the dengue virus) or vaccination in the past against yellow fever or Japanese encephalitis [52, 53].

**Fig. 1:**
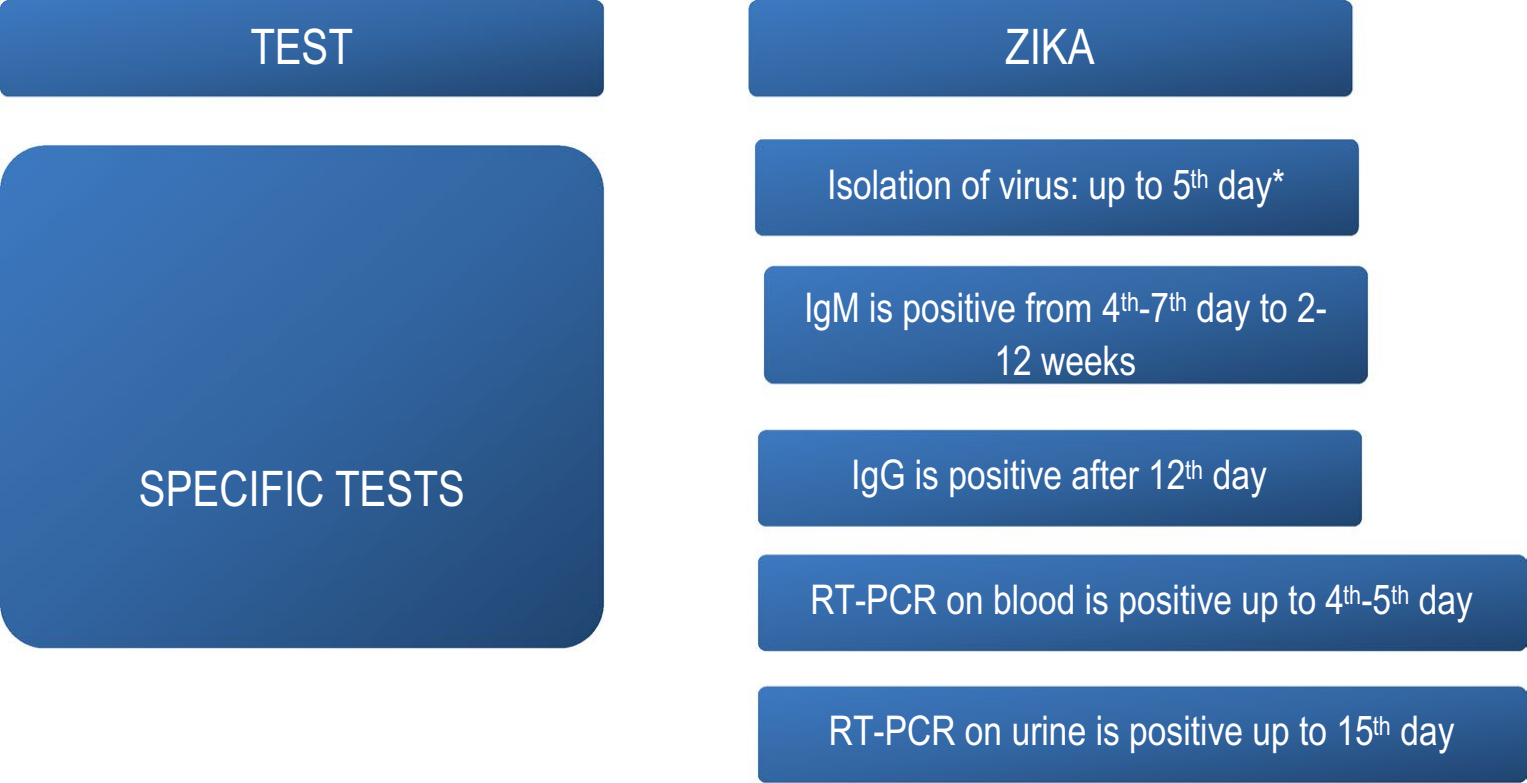
Summary of the recommendations for specifically diagnosing Zika

Negative serological results (non-reactive IgM and IgG) suggest that infection did not occur, if the test was done between 2 and 12 weeks after the exposure [53]. Figure 1 shows the summary of the recommendations for laboratorial diagnosis of zika virus infection. Tables 3, 4 and 5 show the recommendations for collecting, storing, conserving and transporting serological samples, virus isolation and molecular diagnosis in suspected cases of Zika.

**Table 3:** Guidelines for collecting, storing, conserving and transporting serological samples, virus isolation and molecular diagnosis in suspected cases of Zika

Type of diagnosis	Type of material	Collection procedure	Storage and conservation	Packing and transportation
Serological tests	Serum	Collect around 10 ml of blood from the adult, without anticoagulant: first collection 3–5 days after the start of symptoms and second collection 3–4 weeks later. Separate out at least 2–3 ml of serum for serological tests	Use sterile plastic tube with screw top and sealing ring. Label the tube with the patient’s name, collection date and sample type. *Conserve in freezer at *−20° C	Pack in biological sample transportation box (category B UN/3373) with recyclable ice
	Cerebrospinal fluid	Collect 1 ml	Use sterile plastic tube with screw top and sealing ring. Label the tube with the patient’s name, collection date and sample type. *Conserve in freezer at *−20° C	Pack in biological sample transportation box (category B UN/3373) with recyclable ice
RT-PCR	Blood/serum	Collect around 10 ml of blood, without anticoagulant, 3–5 days after the start of symptoms. Separate out at least 2–3 ml of serum, for RT-PCR	Use sterile plastic tube with screw top and sealing ring. Label the tube with the patient’s name, collection date and sample type. *Conserve in freezer at *−20 °C *or* −70 °C *until sending to the laboratory*	Pack in biological sample transportation box (category B UN/3373) with dry ice
	Cerebrospinal fluid	Collect 1 ml	Use sterile plastic tube with screw top and sealing ring. Label the tube with the patient’s name, collection date and sample type. *Conserve in freezer at *−20 °C *or* −70 °C *until sending to the laboratory*	Pack in biological sample transportation box (category B UN/3373) with dry ice
	Urine	Collect 8 ml not more than 8 days after the start of symptoms	Use sterile plastic tube with screw top and sealing ring. Label the tube with the patient’s name, collection date and sample type. *Conserve in freezer at *−20 °C *or* −70 °C *until sending to the laboratory*	Pack in biological sample transportation box (category B UN/3373) with dry ice
	*Instructions for collecting and sending samples for laboratory diagnosis of deaths suspected to be due to Zika*			
	Viscera	Collect 1 cm^3^ of brain, liver, heart, lung, kidney and spleen	Use sterile plastic tube with screw top and sealing ring. Label the tube with the patient’s name, collection date and sample type. *Conserve in freezer at *−20 °C *or* −70 °C *until sending to the laboratory*	Pack in biological sample transportation box (category B UN/3373) with dry ice
Histopathology immunohistochemistry	Viscera	Collect 1 cm^3^ of brain, liver, heart, lung, kidney and spleen	Use sterile flask with screw top containing 10 % buffered formalin. Label the tube with the patient’s name, collection date and sample type. *Conserve at room temperature*	Pack in biological sample transportation box (category B UN/3373) *Without Ice . Conserve at room temperature*

*Source* Brazilian Ministry of Health. Informative Note: Procedures to be adopted for surveillance of Zika virus fever in Brazil, 2016

*Note* In cases of Guillain-Barré syndrome, sample collection should ideally be done before plasmapheresis

**Table 4:** Instructions for collecting and sending samples for laboratory diagnosis—for serological diagnosis

For serological diagnosis			
Type of material	Collection procedure	Storage and conservation	Packing and transportation
Blood (serum)	Collect around 10 ml of blood from the mother, without anticoagulant: first collection 3–5 days after the start of symptoms and second collection 2–4 weeks later. Separate out at least 2–3 ml of serum for serological tests. In the case of the newborn, 2–5 ml of blood (preferably from the umbilical cord), without anticoagulant, and separate out 0.5–1.0 ml of serum for serological tests	Use sterile plastic tube with screw top and sealing ring. Label the tube with the patient’s name, collection date and sample type. *Conserve in freezer at −20 °C*	Pack in biological sample transportation box (category B UN/3373) with recyclable ice
Blood (serum) from umbilical cord	Collect 2–5 ml of blood from the newborn, without anticoagulant, at the time of birth	Use sterile plastic tube with screw top and sealing ring. Label the tube with the patient’s name, collection date and sample type. *Conserve in freezer at −20 °C.*	Pack in biological sample transportation box (category B UN/3373) with recyclable ice
Cerebrospinal fluid	Collect 1 ml from the newborn at the time of birth	Use temperature-resistant sterile plastic tube with screw top and sealing ring. Label the tube with the patient’s name, collection date and sample type. *Conserve in freezer at −20 °C*	Pack in biological sample transportation box (category B UN/3373) with recyclable ice

*Source* Health Surveillance Secretariat of the Ministry of Health “Surveillance and response protocol for occurrences of microcephaly related to Zika virus infection” Brasilia, Federal District, December 2015

**Table 5:** Instructions for collecting and sending samples for laboratory diagnosis—for diagnosis via RT-PCR

For diagnosis via RT-PCR (reverse-transcriptase polymerase chain reaction)			
Type of material	Collection procedure	Storage and conservation	Packing and transportation
Blood (serum)	Collect around 10 ml of blood from the mother, without anticoagulant: up to 3–5 days after the start of symptoms. Separate out at least 2–3 ml of serum for RT-PCR. In the case of the newborn, 2–5 ml of blood (preferably from the umbilical cord), and separate out 0.5–1.0 ml of serum for RT-PCR	Use temperature-resistant sterile plastic tube with screw top and sealing ring. Label the tube with the patient’s name, collection date and sample type. *Conserve in freezer at *−20 °C *or* −70 °C *until sending to the laboratory*	Pack in biological sample transportation box (category B UN/3373) with dry ice
Blood (serum) from umbilical cord	Collect 2-5 ml of blood from the newborn, without anticoagulant, at the time of birth	Use sterile plastic tube with screw top and sealing ring. Label the tube with the patient’s name, collection date and sample type. *Conserve in freezer at *−20 °C *or* −70 °C *until sending to the laboratory*	Pack in biological sample transportation box (category B UN/3373) with dry ice
Cerebrospinal fluid	Collect 1 ml from the newborn at the time of birth	Use temperature-resistant sterile plastic tube with screw top and sealing ring. Label the tube with the patient’s name, collection date and sample type. *Conserve in freezer at *−20 °C *or* −70 °C *until sending to the laboratory*	Pack in biological sample transportation box (category B UN/3373) with dry ice
Urine	Collect 10 ml up to 8 dias days after the start of symptoms	Use sterile plastic tube with screw top and sealing ring. Label the tube with the patient’s name, collection date and sample type. *Conserve in freezer at *−20 °C *or* −70 °C *until sending to the laboratory*	Pack in biological sample transportation box (category B UN/3373) with dry ice
Placenta	Collect 3 × 3 cm from the placenta at the time of birth	Obtain 3 fragments from the placenta (dimensions of 1 cm^3^ each), consisting of non-fixed tissue, and transfer to a temperature-resistant sterile flask with screw top and sealing ring. Label the tube with the patient’s name, collection date and sample type. *Conserve in freezer at *−20 °C *or* −70 °C *until sending to the laboratory*	Pack in biological sample transportation box (category B UN/3373) with dry ice

*Source* Health Surveillance Secretariat of the Ministry of Health “Surveillance and Response Protocol for occurences of microcephaly related To Zika virus infection” Brasilia, Federal District, December 2015

Brazilian Ministry of Health’s guidelines determines to collect samples from the first cases in an area without laboratory confirmation of acute Zika virus disease, from 100 % (all) of the pregnant women with suspected acute Zika virus disease, from 100 % (all) of the deaths suspected to be due to acute Zika virus disease and from 100 % (all) of the patients hospitalized in sentinel units, with neurological manifestations and with suspected previous viral infection (Zika, dengue and chikungunya).

### Interpretation of the results

- A positive molecular test confirms the diagnosis of Zika.
- A negative molecular test does not rule out the possibility of Zika.
- A reactive serological test for Zika may be due to acute infection by the virus, a cross-reaction with other flaviviruses or a result from yellow fever vaccination.

## Case management

### Management of acute zika syndrome

There is no specific antiviral treatment.

The treatment consists of rest, oral hydration and use of medications for symptoms.

Analgesics and antipyretics such as dipyrone and paracetamol.

Antihistamine medications to control itching.

Non-steroidal anti-inflammatory drugs (NSAIDs) should not be used until the diagnosis of dengue has been ruled out [54]. Avoid their use in pregnant women beyond the 32nd week of gestation because of the risk of early closure of the arterial duct.

Avoid use of aspirin in children under the age of 12 years because of the risk of Reye syndrome.

It is important to evaluate differential diagnoses, especially in relation to dengue, because of the greater risk of evolution to severe cases. Treat all cases as dengue until this diagnosis has been ruled out.

### Management of pregnant women

Currently, only limited data on pregnant women infected with the Zika virus are available. The data suggest that pregnant women may become infected with the Zika virus in any trimester. However, the incidence of Zika virus infection among pregnant women is unknown. There is no evidence to suggest that pregnant women are more susceptible to Zika or that they present disease of greater severity than do other individuals [55].

Regarding transmission of the Zika virus, there is evidence of virus transmission from the mother to the fetus during pregnancy and also close to the time of delivery. Since no vaccines or prophylactic medications for avoiding Zika virus infection exist, the Brazilian Society of Infectology (SBI) recommends (in agreement with the Centers for Disease Control and Prevention, CDC) that women in any trimester of pregnancy should consider postponing journeys to areas with transmission of this virus. If a pregnant woman lives in or travels to an area with transmission of the Zika virus, she should protect herself to avoid mosquito bites.

Pregnant women who present symptoms compatible with Zika (including fever, skin eruptions, joint pains and red eyes) need to be given priority for laboratory investigation in order to diagnose Zika virus infection.

Routine prenatal use of serological tests for Zika among pregnant women living in areas with the Zika epidemic is not recommended by the Brazilian Ministry of Health.

### The centers for disease control and prevention (CDC) recommends

- If a laboratory test is positive for Zika or inconclusive, use of serial ultrasound examinations should be considered.
- For pregnant women whose fetus has a confirmed diagnosis of microcephaly, the possibility of amniocentesis should be evaluated from the 15th week of pregnancy onwards.
- Pregnant women in a transmission area should undergo serological tests for Zika at the start of their prenatal care.
- Serological tests should be offered to women in areas without autochthonous transmission if they have a history of travel to an area with known Zika virus transmission and are asymptomatic (i.e. without reports of sickness consistent with Zika). These tests should be performed 2–12 weeks after the trip.

*The Brazilian Federation of Gynecology and Obstetrics Associations (FEBRASGO) recommends the following in cases of pregnant women with clinical manifestations of Zika:*

- Ultrasound examination should be scheduled every month until delivery [56]

*The American Society of Gynecology and Obstetrics recommends the following in cases of pregnant women with serological findings that are positive for Zika or inconclusive, and/or who show symptoms of Zika infection:*

- If, at the time of the symptoms or the serological tests, the pregnant woman has not reached her 20th week of pregnancy, an ultrasound examination should be scheduled every 3 to 4 weeks, starting at the 18th week.
- If, at the time of the symptoms or the serological tests, the pregnant woman is already beyond her 20th week of pregnancy, an ultrasound examination should be scheduled every 3–4 weeks, from the time of diagnosis onwards.

**Fig. 2:**
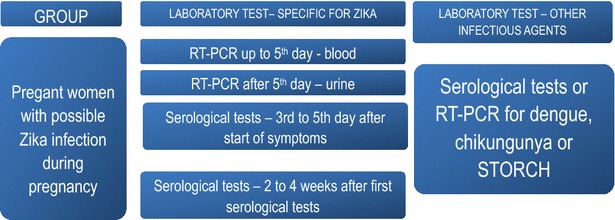
Summary of the recommendations for specifically diagnosing Zika in pregnant women

**Fig. 3:**
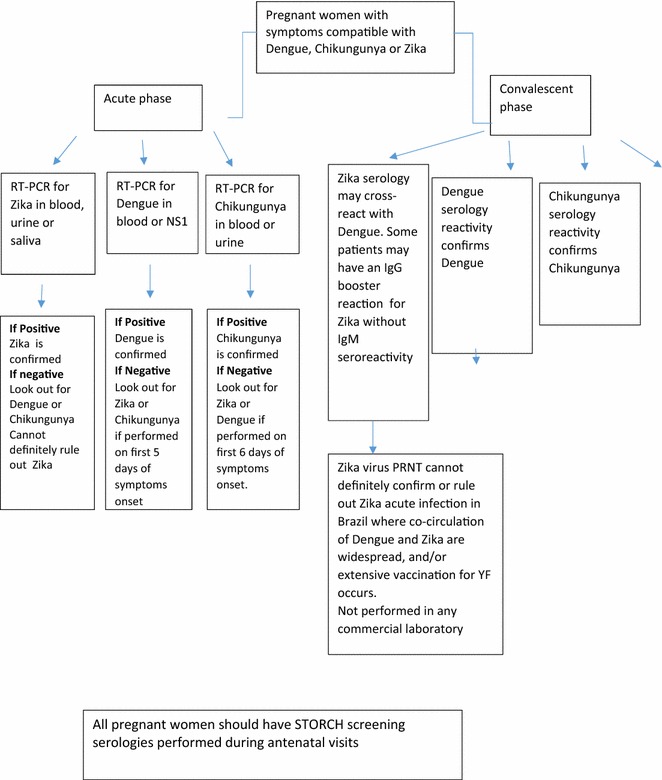
Diagnostic algorithm for different arboviral diseases in pregnant women

The summary of current reccomendations for diagnostic of zika virus infection in pregnant women in shown on Fig. 2. Figure 3 illustrates a diagnostic algorithm for different arboviral diseases in pregnant women.

### Management of microcephaly and congenital malformations

It is very important to record the gestational age at birth when assessing the size of the skull in newborns [57].

The INTERGROWTH-21st allows measuring the head circumference, at birth, across many different populations and it has been obtained through rigorous methodology and standardised procedures. This is very important in the context of the Zika virus outbreak, as reliable information on the head circumference of newborns according to their gestational age is required so as to screen for microcephaly [57].

*Criteria for notification of newborn with microcephaly according to the Ministry of Health’s recommendations [*9*]:*

- Newborns with gestational age of less than 37 weeks that present a head circumference measurement of less than −2 standard deviations for the gestational age and sex, as described in the InterGrowth table.
- Newborns with gestational age of 37 weeks or more that present a head circumference measurement of less than or equal to 31.5 cm for girls and 31.9 for boys, which is equivalent to less than −2 standard deviations for the neonate’s age and sex, as described in the WHO table.

According to WHO and the international literature, microcephaly is defined as a head circumference of less than minus two standard deviations of the reference for the sex, age or length of gestation. The head circumference must be measured using standardized technique and equipment between 24 h and 6 days 23 h after birth (i.e. within the 1st week of life).

Severe microcephaly is defined as a head circumference of less than −3 standard deviations, i.e. more than three standard deviations below the mean for the gestational age and sex.

According to WHO, newborns with microcephaly who present structural abnormalities of the brain, as diagnosed through imaging examinations or observed neurological or developmental abnormalities, should be classified as having “microcephaly with brain abnormality”. All neonates with microcephaly should receive regular evaluations and follow-up during their childhood, including: head growth, the mother’s and family’s histories of pregnancies, evaluation of development and physical and neurological examinations, including evaluation of hearing and eyesight, to identify any problems. To detect structural abnormalities of the brain, WHO recommends that transfontanelle ultrasound examinations should be performed when the size of the fontanelle is sufficient for this procedure. For neonates who present severe microcephaly (−3 standard deviations), cerebral computed tomography or magnetic resonance imaging should be performed.

Microcephaly may be accompanied by epilepsy, cerebral palsy, delayed cognitive, motor and speech development and hearing and eyesight problems.

There is no specific treatment for microcephaly. Since each child develops complications that differ in type and severity, which may include respiratory, neurological and motor problems, the follow-up by different specialists will depend on which functions have been compromised.

**Fig. 4:**
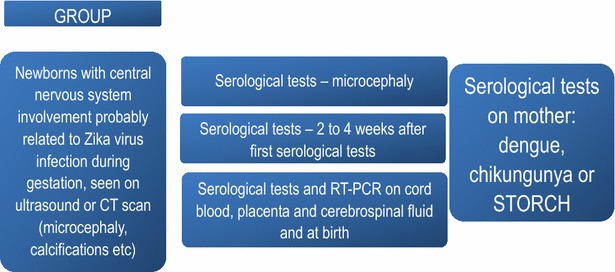
Summary of the recommendations for specifically diagnosing Zika among newborns

The summary of current reccomendations for diagnostic of Zika virus infection in newborns in shown on Fig. 4.

### Management of guillain-barré syndrome

Because of the autoimmune nature of Guillain-Barré syndrome, its treatment in the acute phase consists of immunotherapy, such as plasmapheresis or application of human immunoglobulin. When corticosteroids are used separately, this does not accelerate the recovery or alter the long-term result [58].

The aim of plasmapheresis is to remove the antibodies from the bloodstream and replace them with artificial plasma, usually albumin. The result is better when the procedure is started within the first 7–14 days after the onset of neurological symptoms [58].

Human immunoglobulin accelerates recovery, as observed with plasmapheresis. It is relatively simple to administer. The best results are obtained when it is started within the first 2 weeks after the symptoms begin [58].

Endovenous dose of human immunoglobulin: 400 mg/kg of body weight per day, for a period of 5 days.

Diagnosis of Guillain-Barré syndrome [42]:

- Clinical history and neurological examination.
- Collection of cerebrospinal fluid, in which it is expected to find an increased protein concentration, to the detriment of increased cellularity.
- Patients with suspected syndrome and cellularity greater than 50 cells/mm^3^ should be investigated for other etiologies or for concomitant HIV infection.
- The cerebrospinal fluid may be normal in the hyperacute phase (first week).
- Electroneuromyography is the examination that confirms the diagnosis, but it may be normal in the 1st week.
- Imaging examinations are generally normal.

## Public health control measures

Vector control measures:

- Basic sanitation.
- Elimination of vector foci in homes and common areas.
- Reduction of garbage accumulation through urban cleansing campaigns in areas where garbage collection is irregular, and through increasing the frequency of garbage collection.
- Implementation of vector control through physical, biological and chemical methods, with involvement of families and communities.
- In areas with autochthonous transmission or imported cases of transmission of dengue, chikungunya and/or Zika, blockage of cases through using pesticides targeting adult vectors, primarily through spraying, is recommended, in addition to use of larvicides.
- Fieldwork done by endemic disease agents for controlling both the larvae and the adult mosquitos needs to be monitored and controlled.
- Mosquito control is the only measure that can interrupt the transmission of arbovirus diseases such as Zika, dengue and chikungunya.

## Prevention and personal protection

In order not to infect other people, infected individuals should protect themselves from bites by *Aedes* during the 1st week (viremic phase).

There is still no vaccine for prevention of infection by the Zika virus.

*Steps for preventing mosquito bites*

- Use long-sleeved shirts and long trousers/pants.
- Stay in enclosed places with air conditioning or places with windows and doors that have screens to prevent the entry of mosquitos.
- Sleep under mosquito nets.
- Use registered insect repellents. When these are used as instructed, they are safe and effective, even during pregnancy or breastfeeding.
  - Always follow the guidance of instruction leaflets.
  - Avoid using products that combine repellent and sun protection in the same formulation. The sun protection factor decreases by one-third when used together with insect repellent.
  - If sun protection is used, apply it before applying the repellent.
- For children
  - Do not use repellent on children under the age of 2 months.
  - Dress children in clothes that cover their arms and legs.
  - Cover cots and pushchairs with mosquito nets.
  - Do not apply repellent to children’s hands.
- Clothes impregnated with permethrin can be used.
  - Do not use products containing permethrin directly on the skin.

In Brazil, the National Health Surveillance Agency (ANVISA) only recommends use of repellents for children over the age of 6 months. The Centers for Disease Control and Prevention (CDC) recommends their use from the age of 2 months, except for lemon eucalyptus, which should only be used from the age of 3 years.

## Declarations

We believe that our article “Management of Infection by the Zika Virus” should be published in *Annals of Clinical Microbiology and Antimicrobials* because it is an official document of the Brazilian Infectious Diseases Society and contains the recommendations for managing this infection based on the experience that Brazilian infectious disease specialists gained after facing this epidemic. This document also considers the most recent scientific evidence about zika transmission and clinical features. We intend to submit this article for a special supplement about Arboviruses at *Annals of Clinical Microbiology and Antimicrobials.*

All authors declare no competing interests and confirm that have approved the manuscript for submission. We also confirm that the content of the manuscript has not been published, or submitted for publication elsewhere.

## Abbreviations

WHO: World Health Organization
RNA: ribonucleic acid
PCR: polymerase chain reaction
IgM: immunoglobulin M
RT-PCR: real time polymerase chain reaction
GBS: Guillain-Barré syndrome
NSAID: nonsteroidal anti-inflammatory drug
SBI: Sociedade Brasileira de Infectologia
CDC: Centers for Disease Control and Prevention
FEBRASGO: Federação Brasileira de Ginecologia e Obstetrícia
ANVISA: Agência Nacional de Vigilância Sanitária
